# The brightness dimension as a marker of gender across cultures and age

**DOI:** 10.1007/s00426-019-01213-2

**Published:** 2019-06-14

**Authors:** Carla Sebastián-Enesco, Gün R. Semin

**Affiliations:** 1 Education Sciences, Universidad de La Rioja, Calle Luis Ulloa 2, 26004 Logroño, Spain; 2 William James Center for Research, ISPA - Instituto Universitário, Rua Jardim do Tabaco 41, 1149-041 Lisbon, Portugal; 3 Utrecht University, Utrecht, The Netherlands

## Abstract

Universally, female skin color is lighter than male skin color, irrespective of geographical location. This difference is a distinctive and universal adaptive pattern that emerges after puberty. We address whether this sexual dimorphism is cognitively and culturally represented to ground gender. To this end, we examine a non-Western, non-industrialized population, namely the Wichí (Salta, Argentina) and a Western industrialized population (Spain). The two cultural populations included both adults and prepubescent children. Across two experiments, we utilized a novel task with children and adults who had to make a choice for a female (male) target person between two identical objects that differed only in terms of their brightness. The results in both experiments revealed that the children from the two cultural communities choose a lighter colored object for the female target and a darker version of the same object for the male target. This pattern held across cultures irrespective of the age of participants, except for the male Wichí participants. We discuss how sexual dimorphism in skin color contributes to a universal grounding of the gender category, and advance possible explanations as to why Wichi males did not consistently link gender and brightness.

**Electronic supplementary material:**

The online version of this article (10.1007/s00426-019-01213-2) contains supplementary material, which is available to authorized users.

## Introduction

Few abstract categories, if any, are marked unambiguously, both objectively and subjectively. Sex and gender, however, aside from their polymorphous cultural and political semantics, constitute precisely such categories. On the one hand, sex is marked, unambiguously and universally, by the *sexual* dimorphism of skin color—an objective, evolved physical reality. Universally, females carry a lighter skin than males (Frost, 1988; Jablonski & Chaplin, 2000). On the other hand, this objective dimorphism is anchored culturally on a brightness dimension in the case of gender, a socially constituted abstract category (e.g., Semin & Palma, 2014; Semin, Palma, Acartürk, & Dziuba, 2018). It is a representation that escapes conscious access (e.g., Carrito & Semin, 2019). Indeed, the anchoring of gender on the brightness dimension appears to be consistent across different cultures such as the Portuguese, Dutch, and Turkish cultures (i.e., Semin & Palma, 2014; Semin et al., 2018).

In short, objectively females have a lighter skin color than males irrespective of geographical location (e.g., Jablonski & Chaplin, 2018) and this difference is a distinctive and universal adaptive pattern (Jablonski, 2004; Jablonski & Chaplin, 2000, 2002). To complement this objective phenomenon, culturally the alignment of female–male with the bipolar dimension of light–dark has been demonstrated across a sample of European cultures. There are two questions that are addressed in the research reported here. The first is: can the grounding of gender on the brightness dimension be generalized to a non-Western, non-industrialized population, namely the Wichí (Salta, Argentina)? The second question we addressed was whether a sample of prepubescent children aged 6 to 9 years has the same grounding of gender on a brightness dimension? This age range is significant because it is well known that the sexual dimorphism of skin color emerges only after puberty and is not a feature prominent in the younger age groups that we targeted (see Robins, 1991). To address these questions, we conducted two studies with children and adults, namely one with a Wichí sample and one with a Spanish sample.

In the following, we start with a brief overview of the literature on the sexual dimorphism of skin color. We then turn to the research on the association between gender and brightness and then present an overview of the current research.

### Sexual dimorphism in skin color as a result of natural selection

It is only recent that the difference in skin coloration between males and females has been objectively established and that the evolutionary bases as well as the adaptive function of this dimorphism have been systematically examined. The data based on analyses of unexposed skin areas collected in all the populations that have been studied indicate that women have a lighter skin than men (Jablonski, 2013; Jablonski & Chaplin, 2010), ruling out the possible argument that outdoor time budget can account for the differences between males and females. Moreover, this difference holds across latitudes, namely it is orthogonal to geographical variations in skin pigmentation.

A variety of arguments have been advanced to account for this phenomenon. It has been argued that the sexual dimorphism in skin color is the result of *sexual* selection, either because men preferentially select lighter female skin, or as a product of sexual competition between females, or even to reduce male aggressiveness. It is, however, the hypothesis advanced by Jablonski and Chaplin (e.g., 2000) that has garnered considerable empirical support. According to these authors, sexual dimorphism is primarily driven by *natural* selection (see also Madrigal & Kelly, 2007). Thus, skin pigmentation is an adaptive trait resulting from the tradeoff between protection against ultraviolet radiation (UVR) (provided by darker skin tones) and production of vitamin D (enhanced by lighter skin tones). Females need to maximize the synthesis of vitamin D during pregnancy and lactation to increase their infant’s and their own biological fitness. Therefore, they benefit from having a lighter skin. In contrast, darker skin pigmentation may have been the result of natural selection in males as it optimizes the folate levels in body, which in turn safeguard sperm production. “Because folate is essential for the synthesis of DNA in dividing cells, anything that involves rapid cell proliferation, such as spermatogenesis (the production of sperm cells), requires folate” (Jablonski & Chaplin, 2002, p. 170). Thus, sexual dimorphism in skin color is adaptive for both females and males.

Interestingly, sex differences in skin color emerge late in development (Jablonski, 2012; Paik et al., 2012; Robins, 1991). Girls and boys are born with similar skin pigmentation. Through puberty, the skin of children progressively darkens with no differences between sexes prior to adolescence. Around the ages of 14–16, the skin lightens for both sexes, but the lightening process is more accentuated in females, producing the sex difference in skin color by the end of adolescence (e.g., Kalla & Tiwari, 1970; Mesa, 1983). These developmental trends in skin pigmentation are driven by sex hormones (Jablonski, 2013; Robins, 1991).

### Conceptions of male and female skin color

Inspired by Jablonski’s work (e.g., 2012), Semin and his colleagues have investigated the cognitive representation of the sexual dimorphism in skin color. Do people have an explicitly accessed representation of the difference in skin color between males and females? When participants are asked about the nature of the male–female skin color, then the majority claim that there is no difference in skin color between males and females (Carrito & Semin, 2019). Nevertheless, their performance on a variety of tasks demonstrates that differences between the genders in skin color implicitly drive their performances on a variety of implicit cognitive tasks.

For instance, in a set of speeded classification tasks, Portuguese and Dutch adults were shown to process masculine names faster when they were presented in dark colors as compared to light colors, and the reverse was true for feminine names (e.g., Semin & Palma, 2014). Moreover, when presented with unreadable names in the form of black and white blobs, a sample of Portuguese participants was significantly more likely to classify them as males when they came in black and as females when they came in white, even if they were not able to identify a name at all (Semin et al., 2018). The dimension of brightness not only drives the categorization of females and males, but also the attribution of gender-based preferences. In a choice study presenting a series of commercial objects, Dutch participants inferred females’ and males’ commercial preferences on the basis of the objects’ brightness, namely they preferentially matched the dark object with the male character and the light object with the female character (Semin & Palma, 2014). In a similar experiment with Turkish adults using visual inspection as the dependent measure, they found that participants looked longer and made more eye fixations on dark objects when choosing for a male and vice versa for a female (Semin et al., 2018). Finally, it was found that individuals not only expected female faces to be lighter than male faces but also were more likely to recall female faces as lighter and male faces as darker than originally presented, evincing the impact of the brightness–gender categorization on recognition memory (Carrito & Semin, 2019).

The research reported so far has been conducted with Western Industrial population samples and with adults. We know, however, that sex differences in skin color emerge late in development. It is only during puberty that the sexual dimorphism of skin color emerges raising the question if prepubescent children have the same implicit representation as adults. A second and equally important question is whether the same gender marking of skin color can be found in a non-industrialized culture. It is to these questions that we turned in the research we report below.

## Overview and rationale

### The questions

The first of the two questions addressed in this paper was: would children display the same pattern of gender skin color representation as the one found for adults? As we mentioned earlier, the sex discrepancy in skin color between males and females arises only by the end of adolescence (e.g., Robins, 1991). Therefore, prepubescent children may not have enough observational material to form a representation that grounds gender by brightness, as they can only extract regularities from adult females and males, but not from their peers. Consequently, one could expect to find a weaker gender–brightness association in children as compared to adults. We, therefore, decided to examine prepubescent children and expected that a sample of children between the ages of 6 and 9 would be a plausible one to track the ontogenetic origins of the gender representations before the human skin tone starts its sexual differentiation.

The second question addressed the generality of the findings to date. The cultures from which the research samples came were Dutch, Portuguese and Turkish. These cultural groups share some common grounds, such as skin color, but also access to each other’s cultural practices and historical backgrounds. Crucially, in industrialized populations, such as the ones explored so far, the observational material available to extract skin color regularities is not confined to the surrounding males and females, but is also accessible through the media, as differences in skin color are also represented in books, TV and other cultural products. Would the data obtained from these samples generalize to non-industrialized, small-scaled populations where individuals have a darker skin color (Robins, 1991), and whose media exposure to other male and female exemplars is limited?

To examine the grounding of gender with brightness and maintain a comparative basis, we sampled both younger participants and an adult sample in Spain and in a small-scale South American population, the Wichí of Northern Argentina.

### The choice of the samples: a non-industrial culture and an industrial one

We selected a contrasting pair of samples. The first is the Wichí, who live in the Gran Chaco region in Northern Argentina and Southern Bolivia. Traditionally, the Wichí were semi-nomadic and subsisted on hunting, fishing, gathering, honey collection, and seasonal “slash-and-burn” horticulture (Miller, 2001). Most Wichí are now sedentary and live in often impoverished communities. Apart from governmental assistance for families, their subsistence depends on gathering, selling art craft, and sometimes wage labor. Sexual division of labor is clear cut (Arenas, 2003; Von Koschitzky, 1992). Women’s occupations are domestic chores, gathering, and knitting patterned bags (“yica” or “hilu”) together with other female members (Palmer, 2005), while men are usually in charge of woodworking.

In the Wichí community, children younger than 6 are not expected to contribute to the family economy, and they are usually allowed to play freely. As they grow older, they progressively begin to participate in their parents’ activities. Gender differentiation starts around the age of 6 among the Wichí, with girls and boys using different social artifacts (clothing, games), and different social spaces (Montani, 2012). Girls gradually start to cooperate with females and elders of both genders in the household work, such as house cleaning and younger siblings’ care. In contrast, boys begin to step away from the domestic space, and start to be part of the male tasks such as field trips (Montani, 2008, 2012). Children start primary school at the age of 6, where they are taught in Spanish. Although most Wichí children know what a computer is (i.e., there are occasional computer lessons at school) and how mobile phones work (i.e., adults usually have one), they rarely have access to the Internet, books, or TV. Therefore, their media exposure is very limited.

The second sample was a typical Spanish one of children and adults from Madrid, which were included for two reasons, namely, to check the robustness of the earlier findings in industrialized populations with a new Western population, and to compare data from the Wichí children with Spanish children.

### The experimental paradigm

The task in the experimental paradigm required children and adults to respond to a classification task in which the drawing of a female (male) face was presented first. Then, they were asked to choose the object that they thought belonged to the person by pointing to one of the two objects. The two objects differed in brightness, namely light and dark (or white and black) and were taken from the NOUN database (Novel Object and Unusual Names, Horst & Hout, 2016). They were unfamiliar to the participants and, based on a pilot study, they were found not to be gender related.

We expected a developmental trend with children being less likely than adults to align gender with the brightness dimension. Two sets of findings from the implicit learning literature suggest this to be likely. First, some studies in implicit learning report age differences with adults and teenagers performing better than children (e.g., Arciuli & Simpson, 2011; Maybery, Taylor, & O’Brien-Marlone, 1995; however, see Saffran, Newport, Aslin, Tunick, & Barrueco, 1997 for conflicting findings). Second, as mentioned earlier, the sexual dimorphism in skin color is manifested only by the end of adolescence. Therefore, children may not have sufficient observational instances to form a representation of gender grounding by brightness.

Additionally, we considered the possibility of cultural differences in the developmental pattern with Spanish children displaying a more consistent gender–brightness interface as compared to the Wichí children due particularly to the differential media exposure. Regarding adults, we predicted that both Wichí and Spanish participants would display the gender–brightness association, but again we considered that in Spanish adults such differentiation would be likely to be stronger due to media exposure.

## Experiment 1: Spanish subsample

### Methods

#### Participants

A total of 90 individuals participated in this experiment. In line with the second author’s host institution’s ethical research guidelines (ISPA-Instituto Universitário, ref.: 824153), participants in both experiments reported here were asked to agree with the informed consent. Participants were assured that all data collected would be treated anonymously and would only be published in scientific outlets and that they were free to withdraw at any time without providing a reason. Across both experiments, the studies were performed in accordance with the Declaration of Helsinki for experimentation with human subjects. The sample of children consisted of *N *= 60 participants (33 girls, mean age= 7.72 years, range= 6.33 to 9.08 years) who attended school and studied 1st, 2nd, or 3rd grades. The adult sample consisted of *N *=30 participants (15 females, mean age= 32.10 years, range= 21.10 to 48.05 years). All the children were recruited from the same school in Madrid (Spain) serving middle SES families and belonged to the Spanish majority ethnic group. After having the agreement of the school, written parental consent was obtained for all children, and only children who wanted to participate took part of the study. The adult participants were all Spaniards who were asked to give their written consent before the start of the study.

#### Stimulus material

**Fig. 1:**
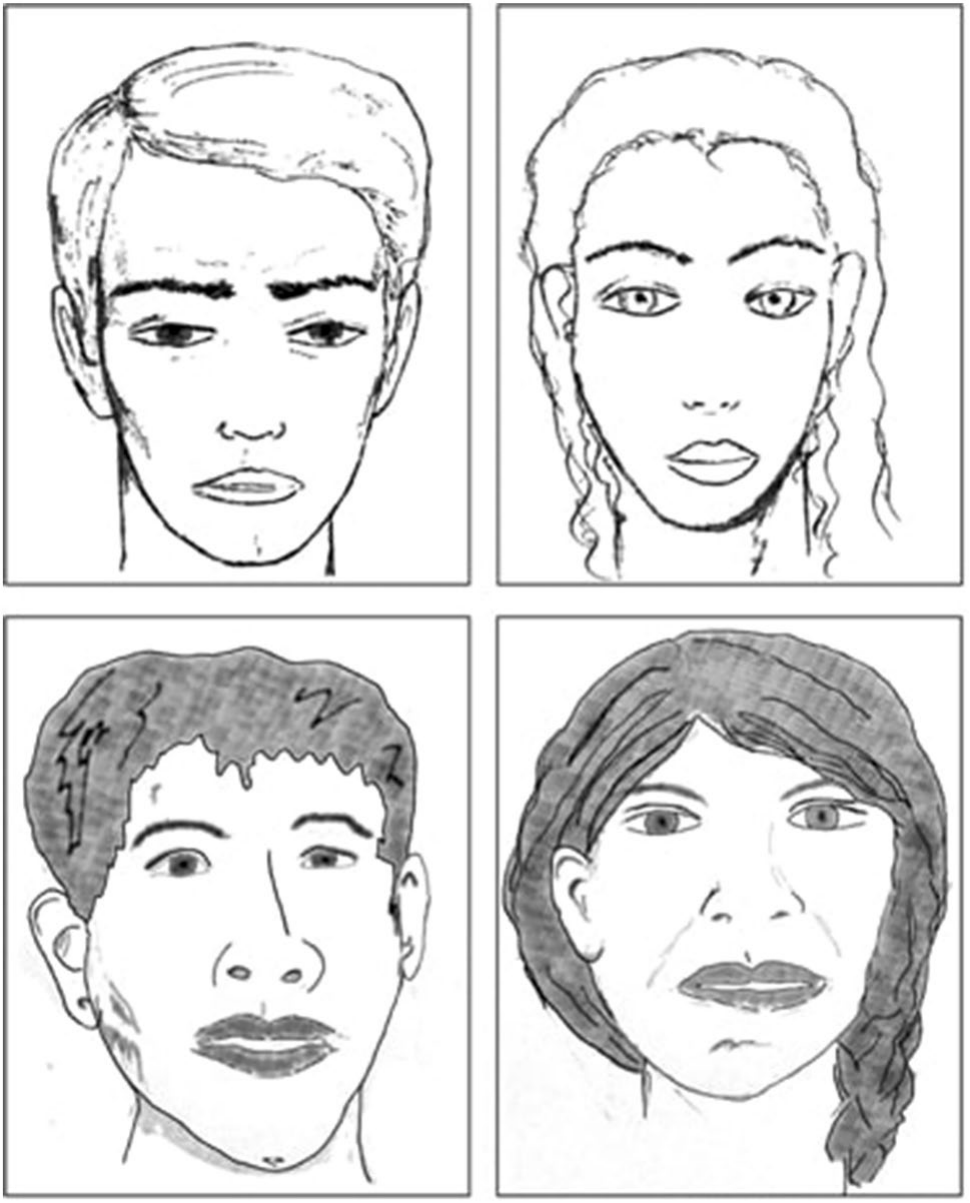
Drawing of male and female faces presented as target characters. The above versions were used in Experiment 1 (Spanish sample) and below versions in Experiment 2 (Wichí sample)

Each trial consisted of first a presentation of a drawing of either a female face or a male face (see Fig. 1). Then, two identical objects that varied on brightness (light and dark) were presented. Each pair of objects was presented twice in two different versions: once in light and dark green as it was a gender-neutral color (hereafter, the green version), and the other time in black and white (hereafter, the monochromatic version), see Fig S1 for examples of the two versions. We administered a total of 18 trials, 9 trials in the monochromatic version and 9 trials in the green version.

#### Procedure

The procedure, presented on a monitor, was identical for the children and the adults. All participants were tested individually in a quiet room. Children were tested in their primary school; adults at their work place. A female experimenter was in charge of all the testing. Participants were first instructed that they would see drawings of different people and different objects and that their task consisted of choosing the object that they thought belonged to the person presented. Each trial consisted of the presentation of a female (male) face drawing on the top of the monitor, and the two objects below at each side (left and right) of the monitor (see SM for a detailed description of the procedure). Participants were asked to choose one of the two objects by pointing to it as fast as they could. Once the experiment was over, the participants were debriefed and thanked for their participation.

#### Data coding and analysis

All responses were coded online by the female researcher. Our main dependent measure was the participants’ object choice in each trial: we coded 0 if participants chose the light object, and 1 if they chose the dark object. First, we tested whether the age of participants in both the adult and children samples influenced their likelihood to align gender with the brightness dimension. For this purpose, participants were given a score for the number of times they assigned light-female or dark-male, out of the 18 trials. We explored the association between age (in years) and the total score using Spearman’s rho correlation.

The main analyses were conducted using generalized linear mixed models (GLMMs) with a binomial distribution and a log link (see SM for full details of the statistical analyses). In all models, we included the participant identity (ID) as a random effect, and trial number for each participant as the random slope to account for the variability across trials within participants. We created separate models for children and adults. In both cases, our main predictor was the target character’s gender (female, male). But we also considered the color version of the objects (green, monochromatic), the participant gender (female, male), and all the 2- and 3-way interactions as fixed effects. For exploratory purposes, we also included the side of the light object (left, right) as a predictor for both adults and children. We used a likelihood ratio test (LRT) to see if the inclusion of predictors improved the model fit.

Finally, we were interested in knowing whether the gender–brightness association was as strong in children as in adults. Therefore, we compared children’s and adults’ total scores using Chi-square tests.

### Results

#### Spanish children

A preliminary analysis indicates that the gender–brightness association was not affected by the age of the children, Spearman rs (60) = .04, *p *= .75. Therefore, subsequent analyses did not consider this variable.

Overall, the full model on children’s object choice provided a better fit to the data than did the null model that only included the random-effect structure (LRT, *χ*^2^= 166.4, *df *= 8, *p *< .001), showing that the predictors potentially explain a part of the data variation. Regarding the sub-model comparisons, the inclusion of side did not improve the model fit (LRT, *χ*^2^= 2.08, *df *= 1, *p *= .15). Therefore, the variable was removed from subsequent models. Subsequent model comparisons showed that none of the interaction terms were significant predictors: three-way interaction (LRT, *χ*^2^= .05, *df *= 1, *p *= .82), character’s gender by color (LRT, *χ*^2^= 1.77, *df *= 1, *p *= .18), participant’s gender by color (LRT, *χ*^2^= .81, *df *= 1, *p *= .37), and participant’s gender by character’s gender (LRT, *χ*^2^= .47, *df *= 1, *p *= .49). The same was true for the main effects of color (LRT, *χ*^2^= 2.80, *df *= 1, *p *= .10) and participant’s gender (LRT, *χ*^2^= .32, *df *= 1, *p *= .57). The only predictor that significantly improved the fit of the model was the target character’s gender (LRT, *χ*^2^= 158.2, *df *= 1, *p* < .001) and, thus, the final model comprised this variable as the only fixed effect (see Table S1).

**Fig. 2:**
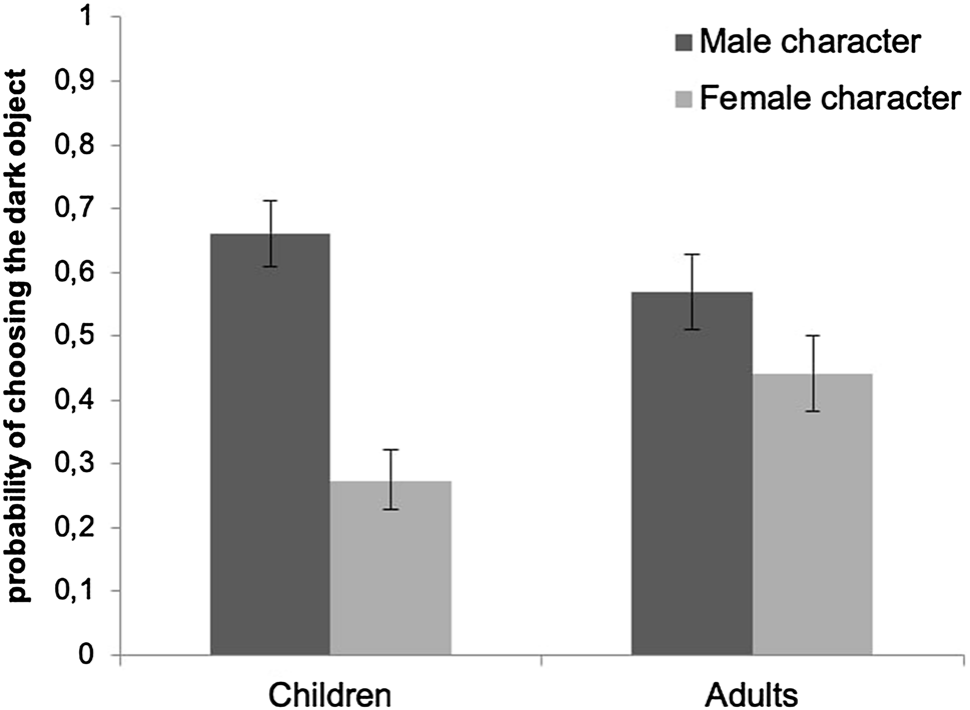
Estimated probability of choosing the dark object based on the GLMMs with character’s gender as predictor, for the children and the adult samples. Error bars are 95% confidence intervals

Children were significantly more prone to choose the dark object when a male was presented (69.70%) than when a female was presented. Likewise, when a female was presented, they were more likely to choose the light object (67.30%), see Fig. 2 for the estimate probabilities. The Fisher’s exact test confirmed this finding, *n *=1080, *p *< .001, Cramer’s *V* = .360. Importantly, the brightness–gender link remained stable across the object color version, green and monochromatic, as the interaction between object color version and the character’s gender was found to be non-significant.

#### Spanish adults

Again, the age of the participants in the adult sample was not related to their likelihood to align gender with brightness, Spearman rs (30) = − .15, *p *= .42.

Regarding the main analyses, we found that the full model provided a better fit than the null model (LRT, *χ*^2^= 14.15, *df *= 6, *p *= .03). As in the case of the children, side was not a significant a predictor of the adults’ likelihood of choosing the dark object (LRT, *χ*^2^ = .32, *df *= 1, *p *= .57). None of the interactions were found to be significant: 3-way interaction (LRT, *χ*^2^= .006, *df *= 1, *p *= .94), color by target character’s gender (LRT, *χ*^2^= .07, *df *= 1, *p *= .78), color by participant’s gender (LRT, *χ*^2^= .72, *df *= 1, *p *= .40), and participant’s gender by target character’s gender (LRT, *χ*^2^= 1.29, *df *= 1, *p *= .26). Likewise, the object’s color (LRT, *χ*^2^= .64, *df *= 1, *p *= .43) and participant gender (LRT, *χ*^2^= 2.72, *df *= 1, *p *= .10) were not significant predictors. Our final model only comprised the target character’s gender as a fixed effect (LRT, *χ*^2^ = 9.15, *df *= 1, *p *= .002), see Table S2.

Spanish adults were significantly more likely to choose the dark object when a male was presented (56.41%) than when a female was presented, see Fig. 2 for the estimate probabilities. Moreover, they preferentially chose the light object when a female was presented (56.55%). As in the case of the children sample, the Fisher’s exact test confirmed this finding, *n *=540, *p *= .003, Cramer’s *V* = .130.

#### Comparison between children and adults

Spanish children were significantly more likely to assign light-female and dark-male (68.43% of trials) than adults (46.48% of trials), *χ*^2^ (2, *N *= 1620) = 72.03, *p *< .001.

### Discussion

In study 1, we found that both Spanish children and adults preferentially assigned dark objects to males and the light objects to females. This gender–brightness pattern was not affected by the color of the objects, presented in either monochromatic shades or green shades, as shown by the lack of a significant main effect of color as well as any interaction effects.

Interestingly, and contrary to our prediction, the gender–brightness pattern was more consistent in children than in adults. Results show that already by the age of 6, children’s representations of gender are consistently grounded with the sensory dimension of brightness. This is true despite the fact that girls and boys of the age range studied have a similar skin tone (Robins, 1991) and, therefore, the “online” observational material available to extract skin color regularities is confined to a relatively limited number of male and female adults. Yet, there is at least one additional source from which children could access the gender–brightness association: The media. It is possible that the acquisition of gender representations does not rely exclusively on direct observations of an “online” environment, but relies potentially also on TV, books, and other cultural products. If this is the case, then by the age of 6, children from industrialized populations such as the Spanish one have already had considerable exposure to examples of the sex dimorphism in skin color.

## Experiment 2: Wichí subsample

### Methods

#### Participants

A total of 90 participants took part in this experiment. Sixty participants were children (30 girls, mean age= 7.46 years, range= 6.08 to 9.17). All child participants attended school and studied 1st, 2nd, or 3rd grades. The adult sample had 30 participants (19 females, mean age = 30.68 years; range = 16 to 67 years). All participants were either born or raised from an early age in the Wichí community (Misión Chaqueña, Salta, Argentina). The experiment was conducted in Spanish, and only individuals who were fluent enough to understand the instructions in Spanish were invited to participate in the experiment. Permission to conduct the research was also obtained from the Ministerio de Educación, Ciencia y Tecnología, Gobierno de la Provincia de Salta, the school head teacher of school 4528 (Misión Chaqueña), and from Misión Chaqueña community representatives. Verbal consent was obtained from all participants (children and adults). Before starting the study, the parents of the child participants were informed about the goals and procedure of our study, and their rights as well as their children’s rights not to participate in and withdraw from the study at any time.

#### Stimulus materials and procedure

Both the stimuli and the procedure were identical to experiment 1 with a single exception: the drawings of female and male faces were adapted to the Wichí facial features (see Fig. 1). Again, children were tested individually in their primary school by a female experimenter and adults in a quiet place close to the school. Once the experiment was over, the participants were debriefed and thanked for their participation.

#### Data coding and analyses

The statistical approach was identical to the one used in Experiment 1.

### Results

#### Wichí children

The age of the children was not related to their general likelihood to align gender with the brightness dimension, Spearman rs (60) = − .15, *p *= .26. Overall, the full model on object choice was significant compared to the null model only including the random effect and slope (LRT, *χ*^2^= 78.23, *df *= 8, *p *< .001). Results from model comparisons showed that the inclusion of side did not provide a better fit to the data (LRT, *χ*^2^= .33, *df *=1, *p *= .57). A second model comparison showed that the 3-way interaction did not improve the model fit (LRT, *χ*^2^= .002, *df *= 1, *p *= .97). Likewise, we found no significant effect for the interactions between the target character’s gender and color (LRT, *χ*^2^= .16, *df *= 1, *p *= .69), and participant’s gender and color (LRT, *χ*^2^= .64, *df *= 1, *p *= .42), as well as the main effect of color (LRT, *χ*^2^= 1.04, *df *= 1, *p *= .31). In other words, children were equally likely to associate gender and brightness with the green and the monochromatic objects. However, the inclusion of the interaction between the participant’s gender and the target character’s gender (LRT, *χ*^2^= 6.28, *df *= 1, *p *= .012) was a significant predictor for children’s likelihood of choosing the dark object. Hence, the final model comprised the two-way interaction (see Table S3).

**Fig. 3:**
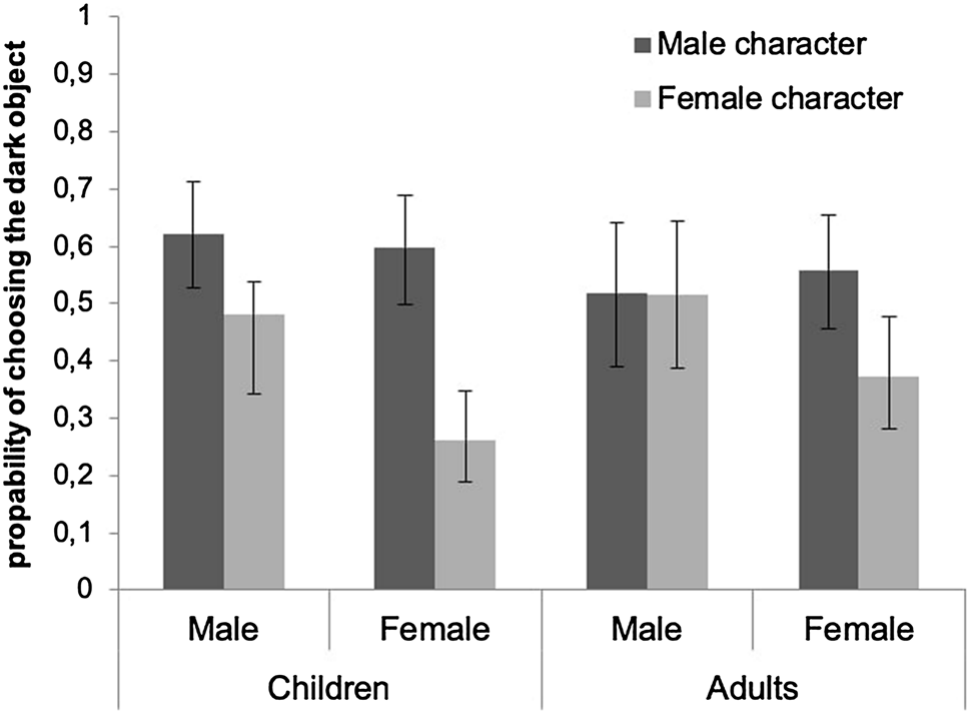
Estimated probability of choosing the dark object based upon the GLMMs with target character’s gender by participant’s gender as predictor, for the children and the adult sample, respectively. Error bars are 95% confidence intervals

Figure 3 displays the estimated rates of choosing the dark object resulting from the interaction of the participant’s gender and the character’s gender. Wichí girls were significantly more likely to choose the dark object when a male target was presented than when a female target was presented. However, boys did not seem to differentiate between male and female targets, as can be seen by the overlapping confidence intervals in Fig. 3. This result was not confirmed by a Fisher’s exact test that showed that both males and females responded differentially to male and female characters (males, *n *=540, *p *< .001; females, *n *= 540, *p *< .001), but that the effect size for the girls (Cramer’s *V* = .306) was larger than for the boys (Cramer’s *V* = .160). Altogether, these findings suggest, at the very least, that for girls the association between gender and their sensory grounding on the light–dark dimension is stronger than that for boys.

#### Wichí adults

The gender–brightness association was not affected by the age of participants, Spearman rs (30) = − .20, *p *= .28. The full model provided a better fit than the null model, meaning that the predictors explained part of the data variation (LRT, *χ*^2^= 21.47, *df *= 8, *p *= .006). Again, side was not a significant predictor of the participants’ likelihood of choosing the dark object (LRT, *χ*^2^= 1.20, *df *= 1, *p *= .27). Likewise, the three-way interaction (LRT, *χ*^2^= 2.04, *df *= 1, *p *= .15) as well as the interaction between target object’s color and the character’ gender (LRT, *χ*^2^= 2.56, *df *= 1, *p *= .11) did not improve the fit of the model. But the inclusion of target object’s color did (LRT, *χ*^2^= 5.87, *df *= 1, *p *= .015), as well as the interaction between the participant’s gender and the target character’s gender (LRT, *χ*^2^= 3.95, *df *= 1, *p *= .047). Therefore, the final model comprised the main effect of target object color and the target gender-by-participant gender interaction term, see Table S4.

Wichí adults were more prone to choose the dark object when presented with the green version than with the monochromatic version. More relevant for our theoretical purposes, males were equally likely to choose the dark object for a female as well as for a male target character, while female participants seemed to differentiate between male and female targets (see Fig. 3 for the estimate probabilities). Although confidence intervals were also overlapping for females, a Fisher’s exact test showed that females were significantly more willing to assign the dark objects to males and light objects to females, females, *n *= 342, *p *= .002, Cramer’s *V* = .161; males, *n *=198, *p *= .38, Cramer’s *V* = .033.

#### Comparison between children and adults

As with the Spanish samples, in comparing the sexual classification patterns in children and adults, we found that children assigned light-female and dark-male significantly more often (61.57% of trials) than did adults (54.81% of trials), *χ*^2^ (2, *N *= 1620) = 6.54, *p *= .011.

### Discussion

The second experiment was designed to examine if members of the Wichí community, a non-industrialized population with limited media access and a skin tone that is significantly darker, choose objects varying in brightness as a function of the gender of the target person for whom they are making the choice. As in experiment 1, we tested Wichí prepubescent children and adults with the same experimental task. The results revealed a gender-related difference in the likelihood to align the target gender with the brightness dimension. Already by the age of 6, girls reliably matched light-female and dark-male. In contrast, boys displayed a less consistent pattern of choices, especially when deciding for female characters, as shown in Fig. 3. Interestingly, this gender difference is strengthened among the Wichí adults, with women revealing the predicted pattern, while men’s choice reflected a chance outcome. Taken together, these findings suggest that the gender representations are anchored in the dimension of brightness in Wichí, but unlike the other populations studied so far, this pattern is confined to females. Finally, as in the Spanish sample, we also found a significant difference between children and adults, with children presenting a stronger gender–brightness link than adults.

## General discussion

Previous research established a consistent interface between the gender category and the brightness dimension in adults from three different industrialized populations and across a number of different experimental paradigms. Individuals not only classified feminine and masculine items according to their brightness, but also expected females to have lighter skin than males, suggesting that the gender categorization patterns are related to the perception of the sexual dimorphism in skin color. However, to provide stronger evidence for the association between skin color and gender marking, three further perspectives on this association are required: Does the demonstrated gender marking hold across populations with distinctly different (a) skin tones; (b) cultural contexts; and (c) age groups? The current research addresses these questions by relying on a new Western population (Spanish) and non-industrialized population with a darker skin (Wichí) (Robins, 1991), and by exploring the developmental differences of this association between children aged 6 to 9 and adults from the two cultural communities. Crucially, according to the anthropological findings (e.g., Jablonski, 2013), children of these ages are still sexually undifferentiated with respect to their skin color.

In both Spanish and Wichí populations, we found a significant effect of the gender of the target for whom a choice was made, and the type of object chosen. Participants preferentially chose the light object for female characters and the dark object for male characters. This was true for children and adults, but the sexual classification pattern was different in the Wichí and Spanish samples. In the case of Spanish adults, none of the other predictor variables explored were significant, indicating that the interface between brightness and gender emerges regardless of the gender of participants, and the color and position of the objects. This finding extends previous results on other industrialized populations, namely Dutch, Portuguese and Turkish adults. Spanish children showed the same trend with no differences across ages, suggesting that the gender–brightness association is already well established by the age of 6, and remained unchanged, at least, until the beginning of puberty.

In the case of the Wichí participants, an unexpected effect of participant gender was observed. Female participants were more likely to assign light-female and dark-male than were male participants. As a matter of fact, these gender differences were more pronounced with age. In the case of the Wichí sample of children, the interface between gender and brightness was found both in boys and girls but the latter displayed a more consistent pattern than the former. However, in adults, the gender marking seemed to be exclusively driven by the female participants’ choices, with males choosing the light and dark objects at chance level. An account for this pattern of results has to be speculative given that we have no data to drive out potential accounts. One possible reason may be of a methodological nature. The person in charge of testing was a female researcher and could have produced a distorting effect in male participants, such as low levels of engagement with the task triggered by a more suspicious attitude toward a (foreign) female in an authority position. This kind of effect of female researchers in cross-cultural fieldwork has been widely explored some decades ago (e.g., Arendell, 1997; Turnbull, 1986; Easterday, Papademas, Schorr, & Valentine, 1977; Warren, 1988). Although none of these studies was conducted in Wichí populations, it is possible that similar problems had arisen in the current research. Moreover, the males of our study started to display a less defined pattern of choices for the objects by the age when the children in the Wichí communities begin to participate in gender-differentiated activities and spend considerable time with same-sex adults (Arenas, 2003; Montani, 2008, 2012). This may mean an asymmetrical exposure to adult males and females for boys, who step away from the domestic space occupied by females and elders of both sexes, and before puberty, start to be part of the male tasks such as field trips (Montani, 2008, 2012). This could explain why Wichí boys were less likely to align gender with the brightness dimension when presented with female characters as compared to male characters.

Contrary to our predictions, the children from both cultures already displayed a consistent gender–brightness association. One possible account for this finding is that the acquisition of the gender–brightness interface goes back to the earliest formation of gender representations. All known cultural communities have some division of roles and duties based on a person’s gender, and this is reflected in their gender representations (Maccoby, 1988). A large body of research indicates that around the age of 3–4 years, children already show a basic understanding of the sex differences associated with possessions, physical appearance, roles, toys, and activities (for a recent review see Martin & Ruble, 2010). Not only children possess conventional knowledge related to gender by these ages, but they also start to recognize some rudimentary metaphorical associations with the gender category, and attribute abstract features to females (e.g., softness) and males (e.g., hardness) (Leinbach, Hort, & Fagot, 1997; Weinraub et al., 1984). Whether or not these early gender representations are already grounded with brightness is a subject that requires research.

Another implication for the early emergence of the gender–brightness link is related to the sources from which individuals could implicitly exploit regularities regarding the sexual difference in skin color. We have considered two main sources that provide observational material: their surrounding males and females, and the media. Yet, apart from visual observation, individuals can also extract information about their environment from the use of their language, namely their linguistic ecology. Indeed, the linguistic context in which a particular word occurs is a key part of its representation (Casasanto & Lupyan, 2015). As such, the co-occurrence of words related to male-dark and female-light, respectively, could lead to the acquisition of “implicit” knowledge about the gender–brightness relation. The works on artificial grammar learning demonstrated that even infants as young as 10 months old are able to learn the predictive relations between linguistic items with only a brief and passive exposure to the conditional probabilities of occurrence (e.g., Gómez & Gerken, 2000; Reber, 1967; Saffran, 2003). Therefore, it is possible that as soon as children are exposed to linguistic stimuli that encompass the probabilistic association between gender and brightness, they implicitly start to generate differential representations of males and females based on brightness.

Another interesting finding, consistent across cultures, is that children presented a more distinct gender–brightness pattern than did adults. We expected to find the reverse pattern based on the fact that prepubescent girls and boys have still similar skin tone and, therefore, are less likely to extract the sexual discrepancy regularities from their own peers.

While we claim the gender grounding with brightness is universal as it is derived from a universal sexual dimorphism in skin color, culture adds layers of interpretation on the interface between gender and brightness. In many, but not all cultures, white is also associated with purity, chastity, innocence, and black is associated with authority, knowledge, war, and this further contributes to the implicit representations of males and females. It is possible that as individuals progressively enrich their implicit representations of gender throughout development, the interface with brightness becomes less accentuated.

Taken together these findings give partial support to the relation between the sex dimorphism in skin color and the gender grounding with brightness. The interface between gender and brightness was not only found in a previously not studied industrialized population (i.e., Spanish), but also in adults from a non-industrialized population with darker skin (i.e., Wichí), although the latter effect was confined to female adults. Therefore, future research should explore other populations to examine the universality of the gender–brightness link.

One of the central contributions of this research is to the developmental roots of the gender–brightness association. We find that already by the age of 6, children from the two different cultures associate the bipolar light–dark dimension with the female–male category, and they did so in a more consistent way than adults. This suggests that the physical evidence (i.e., sex discrepancy in skin color) obtained via “online” observation or through media exposure is not the only force driving the gender marking acquisition. Conversely, it is likely that the skin color regularities are transduced into linguistic patterns (such as the co-occurrence of words related to female-light and male-dark, respectively) within a particular linguistic community and this could contribute to the acquisition of the gender grounding.

Humans often rely on sensorimotor experiences to ground abstract concepts. For instance, the concept of time as movement is grounded by space but with no invariant reference. Therefore, the spatial representations of time are variable around the world. Thus, the way people structure time in space is affected by cultural artifacts that serve as reference points for movement as, for example, writing direction (e.g., Fuhrman & Boroditsky, 2010). This results in cultural differences in the time–space association and how time is grounded. Likewise, gender is also anchored on a sensory dimension, namely the brightness–darkness. However, the marking of gender presents a special case. In contrast to the space–time association, the marking of gender is probably a universal as males and females as grounding referents (i.e., their skin color) are universally dark and light.

## Electronic supplementary material

Below is the link to the electronic supplementary material.

Supplementary material 1 (DOCX 215 kb)

## Data Availability

The datasets generated during the current study are available from the corresponding author on request.

## References

[CR1] Arciuli J, Simpson IC (2011). Statistical learning in typically developing children: The role of age and speed of stimulus presentation. Developmental Science.

[CR2] Arenas P (2003). Etnografía y alimentación entre los Toba-Ñachilamole#ek y Wichí-Lhuku’tas del Chaco Central (Argentina).

[CR3] Arendell T (1997). Reflections on the researcher-researched relationship: A woman interviewing men. Qualitative Sociology.

[CR4] Carrito, M. L., & Semin, G. R. (2019). When we don’t know what we know—sex and skin color. (under review). 10.1016/j.cognition.2019.05.009 31228668

[CR5] Casasanto D, Lupyan G, Margolis E, Laurence S (2015). All concepts are ad hoc concepts. The conceptual mind: New directions in the study of concepts.

[CR6] Easterday L, Papademas D, Schorr L, Valentine C (1977). The making of a female researcher: Role problems in field work. Journal of Contemporary Ethnography.

[CR7] Frost P (1988). Human skin color: A possible relationship between its sexual dimorphism and its social perception. Perspectives in Biology and Medicine.

[CR8] Fuhrman O, Boroditsky L (2010). Cross-cultural differences in mental representations of time: Evidence from an implicit nonlinguistic task. Cognitive Science.

[CR9] Gómez RL, Gerken L (2000). Infant artificial language learning and language acquisition. Trends in Cognitive Sciences.

[CR10] Horst JS, Hout MC (2016). The Novel Object and Unusual Name (NOUN) Database: A collection of novel images for use in experimental research. Behavior Research Methods.

[CR11] Jablonski NG (2004). The evolution of human skin and skin colour. Annual Review of Anthropology.

[CR01] Jablonski NG (2012). Skin: A natural history.

[CR12] Jablonski NG (2013). Skin: A natural history.

[CR13] Jablonski NG, Chaplin G (2000). The evolution of human skin coloration. Journal of Human Evolution.

[CR14] Jablonski NG, Chaplin G (2002). Skin deep. Scientific American.

[CR15] Jablonski NG, Chaplin G (2010). Human skin pigmentation as an adaptation to UV radiation. Proceedings of National Academy of Sciences.

[CR16] Jablonski NG, Chaplin G (2018). The roles of vitamin D and cutaneous vitamin D production in human evolution and health. International Journal of Paleopathology.

[CR17] Kalla AK, Tiwari SC (1970). Sex differences in skin colour in man. Acta Geneticae Medicae et Gemellologiae.

[CR18] Leinbach MD, Hort BE, Fagot BI (1997). Bears are for boys: Metaphorical associations in young children’s gender stereotypes. Cognitive Development.

[CR19] Maccoby EE (1988). Gender as a social category. Developmental Psychology.

[CR20] Madrigal L, Kelly W (2007). Human skin-color sexual dimorphism: A test of the sexual selection hypothesis. American Journal of Physical Anthropology.

[CR21] Martin CL, Ruble DN (2010). Patterns of gender development. Annual Review of Psychology.

[CR22] Maybery M, Taylor M, O’Brien-Malone A (1995). Implicit learning: Sensitive to age but not IQ. Australian Journal of Psychology.

[CR23] Mesa MS (1983). Analyse de la variabilité de la pigmentation de la peau durant la croissance. Bulletins et Mémoires de la Société d’Anthropologie de Paris.

[CR24] Miller ES (2001). Peoples of the Gran Chaco.

[CR25] Montani R, Hirsch S (2008). Metáforas sólidas del género: Mujeres y tejido entre los Wichí. Mujeres Indígenas en la Argentina: Cuerpo, trabajo y poder.

[CR26] Montani, R. (2012). La construcción material de la persona entre los Wichís del Gran Chaco. In *Actas del I Encuentro Latinoamericano de Investigadores sobre Cuerpos y Corporalidades en las Culturas. Facultad de Humanidades y Artes, Universidad Nacional de Rosario, Rosario, Argentina*. Retrieved from http://red.antropologiadelcuerpo.com/index.php/gt-13-ponencias-publicadas/.

[CR27] Paik, S. H., Kim, H. J., Son, H. Y., Lee, S., Im, S. W., Ju, Y. S., … & Kwon, O. S. (2012). Gene mapping study for constitutive skin color in an isolated Mongolian population. *Experimental and Molecular Medicine, 44*, 241. 10.3858/emm.2012.44.3.020 PMC3317488 22198297

[CR28] Palmer J (2005). La buena voluntad wichí: Una espiritualidad indígena.

[CR29] Reber AS (1967). Implicit learning of artificial grammars. Journal of Verbal Learning and Verbal Behavior.

[CR30] Robins AH (1991). Biological perspectives on human pigmentation.

[CR31] Saffran JR (2003). Statistical language learning: Mechanisms and constraints. Current Directions in Psychology Sciences.

[CR32] Saffran JR, Newport EL, Aslin RN, Tunick RA, Barrueco S (1997). Incidental language learning: Listening (and learning) out of the corner of your ear. Psychological Sciences.

[CR33] Semin GR, Palma TA (2014). Why the bride wears white: Grounding gender with brightness. Journal of Consumer Psychology.

[CR34] Semin GR, Palma T, Acartürk C, Dziuba A (2018). Gender is not simply a matter of black and white, or is it?. Philosophical Transactions of the Royal Society B: Biological Sciences.

[CR35] Turnbull CM, Whitehead TL, Conaway ME (1986). Sex and gender: The role of subjectivity in field research. Self, sex, and gender in cross-cultural fieldwork.

[CR36] Von Koschitzky M (1992). Las telas de Malla de los Wichí/Mataco: Su elaboración, su función y una posible interpretación de los motivos.

[CR37] Warren CAB (1988). Gender issues in field research.

[CR38] Weinraub M, Clemens LP, Sockloff A, Ethridge T, Gracely E, Myers B (1984). The development of sex role stereotypes in the third year: Relationships to gender labeling, gender identity, sex-types toy preference, and family characteristics. Child Development.

