# Supplementary material for: The brightness dimension as a marker of gender across cultures and age
Source: Psychol Res. 2019 Jun 14;84(8):2375–84. doi: 10.1007/s00426-019-01213-2 (PMC7515938; doi:10.1007/s00426-019-01213-2)
Supplement: Supplementary file 1 — Supplementary material 1 (DOCX 215 kb) [file 426_2019_1213_MOESM1_ESM.docx]

Supplementary Material

# Method (experiment 1 and 2)

## Stimulus material

Objects used were taken from the NOUN database (Novel Object and Unusual Names, Horst and Hout 2016), and were novel and gender neutral. Each object was presented two times in two different color versions: light and dark greens (green version), and black and white (monochromatic version), see Fig S1 for an example of the objects used in the two versions. We used a total of 9 objects. For the coloring, we selected from an RGB palette the following codes: light green: 0, 255, 0; dark green: 0, 64, 0; white: 255, 255, 255; black: 105, 105, 105. We used a total of 18 face drawings: 9 female faces and 9 male faces that appeared twice, once for the monochromatic trials, and the other time for the green trials. Importantly, we varied the female-male pairs for the monochromatic and green trials. Therefore, a particular female appeared with a particular male in the monochromatic trial but with a different male in the green trial. The female and male faces presented belonged to the same ethnic group as the participant, either Spanish (experiment 1) or Wichí (experiment 2). Crucially, there were no differences in skin color between the male and female faces in the drawings (see Fig 1).


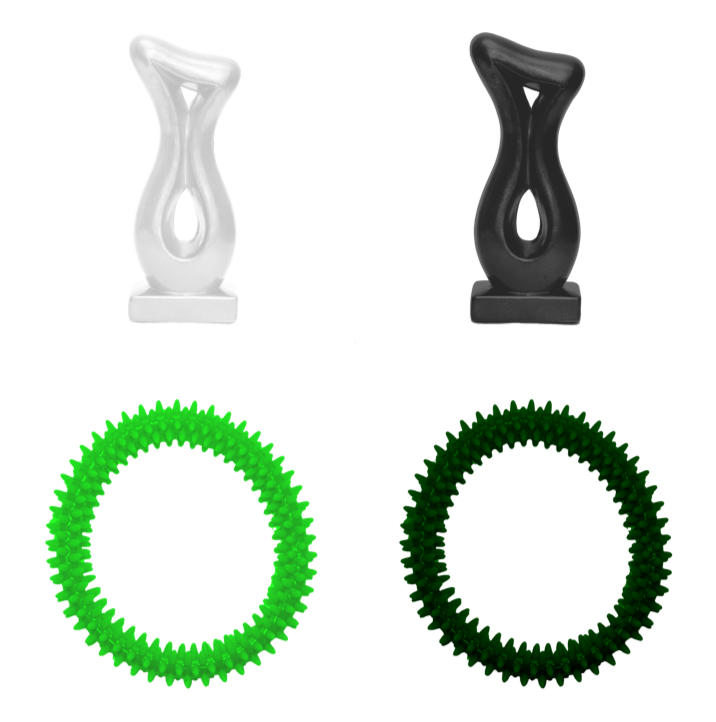


**Fig. S1** Example of the pair of objects used from the NOUN database (Horst and Hout 2016) in the monochromatic version (above) and the green version (below).

## Procedure (detailed)

Participants were first instructed that they would see drawings of different people and different objects and that their task consisted of choosing the object that belonged to the person presented. Immediately after, the experiment began. The sequence of events for each trial was as follows: participants first saw a pair of same-sized drawings of a male and a female faces, side by side; followed by the presentation of one of the drawings in reduced side and the other in enlarged size; then, only this latter drawing was presented; finally, the participants could see the larger picture on the top of the screen and the two objects below at each side (left and right) of the screen. Participants were then asked to choose one of the two objects as fast as they could. The order of presentation was counterbalanced across trials regarding (1) character’s gender (male and female); (2) side of light object (right and left); (3) color version (monochromatic and green). However, all participants were presented with the same fixed order of trials.

Instructions

Once the experimenter greeted the participant, she said: “Now, you will see a series of people and different objects. For each person that appears, you will need to choose the object that belongs to that person as fast as you can.” [in Spanish: “Ahora vas a ver una serie de personas y distintos objetos. Para cada persona que aparece, tienes que elegir tan rápido como puedas el objeto que le pertenece.”] Then, for each trial, the experimenter asked: “What’s the object that belongs to this person?” [In Spanish: “¿Cuál es el objeto que pertenece a esta persona?”]

## Data coding and analyses

Main analyses were conducted using generalized linear mixed models (GLMMs) with a binomial distribution (0 = choice of light object; 1 = choice of dark object), and a log link. In all models, we included participant identity (ID) as random effect, and trial number within participant identity as the random slope to account for variability across trials within participants. We proceeded as follows: we first compared a full model which included all predictors with a null model which only comprised the random effect and slope as explanatory variables. Secondly, we created sub-models from the full model by sequentially dropping single terms (first the exploratory variable, and then the main predictors in order of decreasing complexity – the three-way interaction, two-way interactions, and then main effects), and by testing if their inclusion significantly improved the model fit using likelihood ratio tests, LRTs. This procedure has been shown to be a good method for protecting against type I error rates (Forstmeier and Schielzeth 2011). All statistical analyses were implemented in R (version 3.44; R Core Team, 2016). Mixed models were built using the package “lme4” (Bates Mächler Bolker and Walker 2014).

# Results (experiment 1 and 2)

## Experiment 1: Spanish subsample

### Spanish children

**Table S1.** Estimated effects (and standard errors) for generalized linear mixed models (GLMM) predicting Spanish children’s choice of the dark object. Factor base levels were: Character’s gender = Male, Color = Green. Trial was included as continuous variable and was centered.

|  | **Final model** |
| --- | --- |
| Intercept | 0.67 (0.12)^***^ |
| Character’s gender (Female) | -1.65 (0.14)^***^ |
| AIC | 1336 |
| BIC | 1360.9 |
| Log Likelihood | -663.0 |
| Number of observations | 1080 |
| Number of groups: Participant ID | 60 |
| Variance: Participant ID (Intercept) | 0.27 |
| Variance: Trial (Slope) | 0.003 |

^***^*p <* .001, ^**^*p <* .01, ^*^*p <* .05

### Spanish adults

**Table S2.** Estimated effects (and standard errors) for generalized linear mixed models (GLMM) predicting Spanish adults’ choice of the dark object. Factor base levels were: Character’s gender = Male, Participant’s gender = Male, Color = Green. Trial was included as continuous variable and was centered.

|  | **Final model** |
| --- | --- |
| Intercept | 0.28 (0.12)^*^ |
| Character’s gender (Female) | -0.52 (0.17)^**^ |
| AIC | 749.4 |
| BIC | 770.9 |
| Log Likelihood | -369.7 |
| Number of observations | 540 |
| Number of groups: Participant ID | 30 |
| Variance: Participant ID (Intercept) | 0.1 |
| Variance: Trial (Slope) | 0.0001 |

^***^*p <* .001, ^**^*p <* .01, ^*^*p <* .05

## Experiment 2: Wichí subsample

### Wichí children

**Table S3.** Estimated effects (and standard errors) for generalized linear mixed models (GLMM) predicting Wichí children’s choice of the dark object. Factor base levels were: Character’s gender = Male, Participant’s gender = Male, Color = Green. Trial was included as continuous variable, and was centered.

|  | **Final model** |
| --- | --- |
| Intercept | 0.50 (0.21)^*^ |
| Character’s gender (Female) | -0,75 (0.19)^***^ |
| Participant’s gender (Female) | -0.11 (0.29) |
| Character’s gender (Female) x Participant’s gender (Female) | -0.69 (0.27)^*^ |
| AIC | 1375.4 |
| BIC | 1410.2 |
| Log Likelihood | -680.7 |
| Number of observations | 1080 |
| Number of groups: Participant ID | 60 |
| Variance: Participant ID (Intercept) | 0.72 |
| Variance: Trial (Slope) | 0.0013 |

^***^*p <* .001, ^**^*p <* .01, ^*^*p <* .05

### Wichí adults

**Table S4.** Estimated effects (and standard errors) for generalized linear mixed models (GLMM) predicting Wichí adults’ choice of the dark object. Factor base levels were: Character’s gender = Male, Participant’s gender = Male, Color = Green. Trial was included as continuous variable, and was centered.

|  | **Final model** |
| --- | --- |
| Intercept | 0.29 (0.28) |
| Color (Black) | -0.44 (0.18)^*^ |
| Character’s gender (Female) | -0.003 (0.30) |
| Participant’s gender (Female) | 0.16 (0.34) |
| Character’s gender (Female) x Participant’s gender (Female) | -0.74 (0.37)^*^ |
| AIC | 737.5 |
| BIC | 771.8 |
| Log Likelihood | -360.7 |
| Number of observations | 560 |
| Number of groups: Participant ID | 30 |
| Variance: Participant ID (Intercept) | 0.31 |
| Variance: Trial (Slope) | 0.016 |

^***^*p <* .001, ^**^*p <* .01, ^*^*p <* .05

# References

Bates, D., Mächler, M., Bolker, B., & Walker, S. (2014). Fitting linear mixed-effects models using lme4. *Journal of Statistical Software, 67*, 1-48. doi:10.18637/jss.v067.i01.

Forstmeier, W., & Schielzeth, H. (2011). Cryptic multiple hypotheses testing in linear models: overestimated effect sizes and the winner's curse. *Behavioral Ecology and Sociobiology*, *65*, 47-55. <https://doi.org/10.1007/s00265-010-1038-5>

Horst, J. S., & Hout, M. C. (2016). The Novel Object and Unusual Name (NOUN) Database: A collection of novel images for use in experimental research. *Behavioral Research Methods*, *48, 1393.* <https://doi.org/10.3758/s13428-015-0647-3>

R Core Team (2018). *R: A language and environment for statistical computing.* Vienna, Austria: R Foundation for Statistical Computing. Retrieved from: <https://www.R-project.org/>
